# Relationship between emotion regulation skills, resilience, depression and anxiety symptom severity in patients with mood disorders and non-clinical participants: a mediation model

**DOI:** 10.1007/s00406-025-02050-8

**Published:** 2025-07-03

**Authors:** Hyun Seok Do, Jakyung Lee, Hyeona Yu, Chan Woo Lee, Hyun Jung Hur, Jongwook Lim, Hyo Shin Kang, Jungkyu Park, Tae Hyon Ha, Woojae Myung

**Affiliations:** 1 Department of Neuropsychiatry, Seoul National University Bundang Hospital, 82 Gumi-ro 173 beon-gil Bundang-gu, Seongnam-si, Gyeonggi-do 13620 Republic of Korea; 2 Department of Psychology, Kyungpook National University, Daegu, Republic of Korea; 3 Department of Psychiatry, Seoul National University College of Medicine, Seoul, Republic of Korea

**Keywords:** Emotion regulation skills, Resilience, Major depressive disorder, Bipolar disorder, Mediation analysis, Factor analysis

## Abstract

Emotion regulation has been proposed as a key factor contributing to resilience, both of which are associated with the modulation of psychiatric symptoms. This study aimed to estimate the correlations between the adaptive emotion regulation skills, resilience, and the severity of psychiatric symptoms, as well as the mediating role of resilience in the relationship between the adaptive emotion regulation skills and the severity of psychiatric symptoms including depression and anxiety. Adaptive emotion regulation skills were measured using the Emotion Regulation Skills Questionnaire (ERSQ) and analyses were based on the factor structure of the Korean version of the ERSQ identified in this study. Participants included patients with major depressive disorder (*N* = 362), bipolar I disorder (*N* = 129), bipolar II disorder (*N* = 463) and non-clinical participants (*N* = 3,111). Partial correlations, adjusted for sociodemographic variables, were evaluated for the clinical measures. Mediation analyses, controlling for sociodemographic variables, were performed. Exploratory and confirmatory factor analyses were conducted on the ERSQ. Two factors of the ERSQ were identified: “acceptance and understanding” and “tolerance and engagement.” Positive correlations were found between the resilience and each factor of the ERSQ, while both were negatively related to the magnitude of depression and anxiety. Mediation analyses showed significant negative indirect effects of both factors of emotion regulation skills on the severity of depression and anxiety through resilience. These findings indicate that improving emotion regulation skills may help improve resilience and maintain life with alleviated psychiatric symptoms.

**Supplementary Information:**

The online version contains supplementary material available at 10.1007/s00406-025-02050-8.

## Introduction

Emotion regulation is defined as inner or outer mechanism of observing, appraising, and controlling an individual’s emotional dynamics to achieve objectives [1]. A previous study suggested that deficits in emotion regulation are associated with an increased risk of depression recurrence [2]. In addition, young patients with bipolar disorder (BD) or major depressive disorder (MDD) and a history of lifetime suicidal ideation show shortcomings in emotion regulation [3]. Conversely, emotion regulation skills help with managing depressive symptoms [4, 5] and anxiety symptoms [6]. These findings suggest that emotion regulation contribute to the regulation of psychiatric symptoms.

Resilience is the ability to adapt to, manage stressful conditions, and maintain psychological stability [7, 8]. A previous study on patients with bipolar disorder revealed that depressive symptoms are negatively related to resilience and positively related to emotion dysregulation [9]. Furthermore, greater resilience is related to reduced severity of depressive and anxiety symptoms in healthy individuals [10]. Notably, resilience encompasses both state and trait qualities, implying that an individual’s resilience can be changed [11, 12]. Therefore, identifying the factors that influence resilience can help develop interventions to strengthen resilience and, in turn, sustain mental well-being.

Previous studies have suggested that emotion regulation may explain resilience in individuals with depressive and anxiety disorders [13] and cognitive reappraisal enhances resilience in university students [14]. Furthermore, imaging studies have reported that resilience is related to emotional network regions of the brain [15]. Hence, resilience may mediate the relationship between emotion regulation skills and the severity of depression and anxiety symptoms. This mechanism is supported by studies demonstrating the impact of emotion regulation skills on the severity of depression or anxiety through resilience in college students [16–18]. However, it remains unclear whether the mediation model can be applied to patients with mood disorders as well as individuals without such disorders, as previous studies lacked mood disorder profiles of participants. Moreover, previous studies often used measurements with limitations in encompassing the diverse features of adaptive emotion regulation skills (e.g., Emotion Regulation Questionnaire [19]). Meanwhile, to cover various aspects of adaptive emotion regulation skills, the Emotion Regulation Skills Questionnaire (ERSQ) can be used as an effective tool [20]. This self-reported measure was developed based on the Adaptive Coping with Emotions model and incorporates the dynamics of nine adaptive skills such as “awareness” and “tolerance” [21].

In this study, we aimed to investigate how different facets of adaptive emotion regulation skills influence psychiatric symptoms through resilience in patients with mood disorders and in non-clinical participants, using the ERSQ. We initially aimed to explore how factors of ERSQ, resilience, and severity of psychiatric symptoms, specifically depression and anxiety are correlated. Then, we aimed to elucidate the mediating role of resilience in the relationship between the factors of ERSQ and severity of depression and anxiety. As the factor structure of the ERSQ was not investigated in the Korean version, we identified its factor structure and performed correlation and mediation analyses based on these findings.

## Methods

### Participants

Patients with mood disorders (*N* = 954) and non-clinical participants (*N* = 3,111) were included in this study. The patients were diagnosed with major depressive disorder (MDD, *N* = 362), bipolar I disorder (BD1, *N* = 129), or bipolar II disorder (BD2, *N* = 463) based on the Diagnostic Statistical Manual of Mental Disorders, Fifth Edition (DSM-5) [22] and received treatment at the Seoul National University Bundang Hospital between July 2013 and October 2024. Diagnoses were established by board-certified psychiatrists (W.M. and T.H.H.) using structured diagnostic interviews (Mini-International Neuropsychiatric Interview, M.I.N.I.) or case records [23]. Non-clinical participants (NCP) were individuals aged ≤ 80 years with no history of psychiatric disorder diagnoses and were surveyed online between November 2024 to December 2024 by a professional survey company. Sociodemographic characteristics including age, sex, education, marital status, job status, alcohol use status, and smoking status were collected from participants. All data collection procedures were conducted in accordance with the approved guidelines, and ethical approval was obtained from the Institutional Review Board of Seoul National University Bundang Hospital (approval no. B-2205-756-111).

### Measures

#### ERSQ

Emotion regulation skills were measured using the Korean version of ERSQ [24]. The ERSQ scale comprises 27 items (e.g., “I knew what my feelings meant,” “I could tolerate my negative feelings”). Each item is rated on a 5-point Likert-type scale, with higher scores reflecting greater emotion regulation skills. The total score was calculated as the average of all item scores, with scores ranging from 0 to 4. The ERSQ has been validated in German, English, Turkish, Latvian, Spanish, French, Czech, and Japanese versions [20, 25–31]. The original German version of the scale demonstrated good reliability (Cronbach’s *alpha* = *0.90*) [25]. As the psychometric properties in the Korean version of ERSQ had not been studied, its composite reliability was assessed in the results section of this study (*ω* for the total composite *= 0.97*).

#### Connor-Davidson resilience scale (CD-RISC)

Resilience was measured using CD-RISC [32]. The CD-RISC is a self-report scale comprising 25 items rated on a Likert-type scale ranging from 0 to 4, resulting in a total score ranging from 0 to 100. Higher scores indicated better resilience. A previous study validated the Korean version of the scale and demonstrated good reliability (Cronbach’s *α* = *0.93*) [33]. Internal consistency was considered sufficient for our total sample (Cronbach’s *α = 0.95*).

#### Zung self-rating depression scale (Zung-SDS)

Depression symptom severity was measured using the Zung-SDS [34]. The scale comprises 20 items, each rated on a 4-point Likert type scale, with a total score ranging from 20 to 80, reflecting depression symptom severity. Of the 20 items, 10 items were reverse-scored. The Korean version of the scale was validated in a previous study and demonstrated acceptable reliability (Cronbach’s *α = 0.80* in depressed patients; *0.79* in non-depressed controls) [35]. Internal consistency was considered sufficient for our total sample (Cronbach’s *α = 0.91*).

#### Beck anxiety inventory (BAI)

Anxiety symptom severity was measured using the BAI [36], a self-report scale. The BAI comprises 21 items rated on a 4-point Likert-type scale, with a total score ranging from 0 to 63 with higher scores reflecting higher anxiety symptom severity. A previous study validating the Korean version of the scale reported an internal consistency of *0.91* [37]. Internal consistency was considered sufficient for our total sample (Cronbach’s *α = 0.95*).

### Statistical analysis

Sociodemographic data were analyzed using one-way analysis of variance without assuming equal variances for continuous variables and the chi-square test for categorical variables. Subsequently, post-hoc analyses were conducted using pairwise t-tests (assuming unequal variance) and pairwise chi-square tests.

Factor analyses were conducted to explore the factor structure of the Korean version of the ERSQ scale. The full sample, comprising both the patient group and the NCP was randomly split into two subsamples. Exploratory factor analysis (EFA) was performed on the first subsample. The appropriate number of factors was determined based on the following indicators: inspection of the scree plot [38]; eigenvalues ≥ 1; the root mean square of the residual (RMSR), smaller value indicating better fit of the model and values < 0.08 considered as good fit; whether the factors were clearly interpretable. Given the ordinal nature of the items, a polychoric correlation matrix and weighted least square (WLS) estimator were used for EFA. Moreover, promax rotation [39] was applied, considering the inter-factor correlations. Furthermore, we conducted EFA separately on the whole patient group and the NCP in the first subsample, and calculated congruence coefficients to assess the structural similarity of ERSQ across the groups [40].

Based on the EFA solution, confirmatory factor analysis (CFA) was conducted on the second subsample using the Weighted Least Squares Mean and Variance Adjusted (WLSMV) estimator to validate the factor structure. In the EFA solution, items with factor loadings < 0.5 or those that cross-loaded onto multiple factors were dropped, and model modifications were conducted based on the modification indices (MIs). To assess the goodness of fit, robust Comparative Fit Index (CFI), robust Root Mean Square Error of Approximation (RMSEA), and Standardized Root Mean Square Residual (SRMR) were estimated. Robust CFI > 0.9, robust RMSEA < 0.1, and SRMR < 0.08 were considered indicators of an acceptable fit [41, 42]. Additionally, to test the reliability of the factors, McDonald’s *ω* values were calculated [43].

Partial correlation values were calculated to explore the relationships between the variables while controlling for potential confounders, including age, sex, education, marital status, job status, alcohol use status, and smoking status. The analyses were conducted on the full sample as well as separately for each subgroup (MDD, BD1, BD2, and NCP). Bonferroni-adjusted *p*-values were calculated for post-hoc analyses for each sociodemographic variable (six comparisons per variable), and each partial correlation plot $\:{(N}_{variable}\times\:\left({N}_{variable}-1\right)\div 2)$ [44].

Mediation analyses were conducted to estimate the mediating effect of resilience on the relationship between emotion regulation skills and severity of depression and anxiety for the full sample and each subgroup, employing the robust maximum likelihood estimator (MLR). In addition to the confidence interval based on standard errors estimated via the MLR estimator, 95% percentile bootstrap confidence intervals were calculated using 10,000 draws. The mediation models were adjusted for sociodemographic variables including age, sex, education, marital status, job status, alcohol use status, and smoking status. The analyses were conducted separately for the total score and subscales of the ERSQ, as well as for the Zung-SDS and BAI. R (version 4.4.2) was used for the statistical analyses [45]. Specifically, the EFA and congruence coefficient calculations were conducted with the “psych” package version 2.4.12 [46]. Partial correlations were calculated using the “ppcor” package version 1.1 [47]. CFA and mediation analyses were performed with the “lavaan” package version 0.6.19 [48]. When computing bootstrapped confidence intervals, the “semhelpinghands” package version 0.1.12 was used [49]. McDonald’s *ω* was estimated by “semTools” package version 0.5.6 [50]. Statistical significance was set at *p* < 0.05.

## Results

### Sociodemographic characteristics

Among the 4,065 participants, 362 patients were diagnosed with MDD, 129 with BD1, 463 with BD2, and 3,111 were NCP. As shown in Table 1, among the patient groups, individuals with BD2 were more likely to be younger, smokers and unmarried compared to patients with MDD. Table 1 also reveals that patients with BD1 had significantly higher total ERSQ and CD-RISC scores and significantly lower Zung-SDS and BAI scores than the other patient groups.

**Table 1:** Sociodemographic data

Characteristics		Patient groups				Post-hoc (*p*-value)					
	**NCP** (*N* = 3,111)^*a*^	**MDD** (*N* = 362)^*a*^	**BD1** (*N* = 129)^*a*^	**BD2** (*N* = 463)^*a*^	*p*-value^*b*^	NCP vs. MDD	NCP vs. BD1	NCP vs. BD2	MDD vs. BD1	MDD vs. BD2	BD1 vs. BD2
Sex (Female)	1,971 (63.4%)	244 (67.4%)	87 (67.4%)	333 (71.9%)	< 0.01	≥ 0.05	≥ 0.05	< 0.01	≥ 0.05	≥ 0.05	≥ 0.05
Patient age	38.6 (12.3)	37.7 (13.1)	34.6 (12.3)	31.5 (11.1)	< 0.001	≥ 0.05	< 0.01	< 0.001	≥ 0.05	< 0.001	≥ 0.05
Education (After high school graduation)	2,719 (87.4%)	255 (70.4%)	105 (81.4%)	349 (75.4%)	< 0.001	< 0.001	≥ 0.05	< 0.001	≥ 0.05	≥ 0.05	≥ 0.05
Job status (Employed)	2,347 (75.4%)	147 (40.6%)	48 (37.2%)	178 (38.4%)	< 0.001	< 0.001	< 0.001	< 0.001	≥ 0.05	≥ 0.05	≥ 0.05
Marital status (Married)	1,391 (44.7%)	163 (45.0%)	41 (31.8%)	136 (29.4%)	< 0.001	≥ 0.05	< 0.05	< 0.001	≥ 0.05	< 0.001	≥ 0.05
Alcohol use status (Current drinker)	1,808 (58.1%)	174 (48.1%)	65 (50.4%)	225 (48.6%)	< 0.001	< 0.01	≥ 0.05	< 0.001	≥ 0.05	≥ 0.05	≥ 0.05
Smoking status (Current smoker)	489 (15.7%)	76 (21.0%)	37 (28.7%)	140 (30.2%)	< 0.001	≥ 0.05	< 0.001	< 0.001	≥ 0.05	< 0.05	≥ 0.05
ERSQ	2.3 (0.7)	1.7 (0.7)	2.0 (0.8)	1.8 (0.7)	< 0.001	< 0.001	< 0.05	< 0.001	< 0.01	≥ 0.05	< 0.01
ERSQ (revised)	2.3 (0.7)	1.7 (0.7)	2.1 (0.9)	1.8 (0.8)	< 0.001	< 0.001	< 0.05	< 0.001	< 0.01	≥ 0.05	< 0.01
Awareness and understanding	2.2 (0.7)	1.8 (0.8)	2.1 (0.9)	1.9 (0.8)	< 0.001	< 0.001	≥ 0.05	< 0.001	< 0.05	≥ 0.05	≥ 0.05
Tolerance and engagement	2.2 (0.8)	1.4 (0.9)	1.8 (1.0)	1.3 (0.9)	< 0.001	< 0.001	< 0.001	< 0.001	< 0.001	≥ 0.05	< 0.001
CD-RISC	62.2 (16.0)	44.9 (18.0)	56.1 (20.9)	42.8 (19.2)	< 0.001	< 0.001	< 0.01	< 0.001	< 0.001	≥ 0.05	< 0.001
Zung-SDS	40.9 (8.8)	54.5 (10.3)	45.7 (11.3)	54.9 (9.6)	< 0.001	< 0.001	< 0.001	< 0.001	< 0.001	≥ 0.05	< 0.001
BAI	8.2 (8.6)	22.2 (13.2)	15.6 (14.0)	23.9 (14.1)	< 0.001	< 0.001	< 0.001	< 0.001	< 0.001	≥ 0.05	< 0.001

ERSQ, Emotion Regulation Skills Questionnaire; ERSQ (revised), The ERSQ total score, recalculated with items removed according to the results of the factor analysis; CD-RISC, Connor-Davidson Resilience Scale; Zung-SDS, Zung Self-rating Depression Scale; BAI, Beck Anxiety Inventory; BD, bipolar disorder; MDD, major depressive disorder; NCP, non-clinical participants; ^*a*^n (%), Mean (SD); ^*b*^Pearson’s Chi-squared test, One-way analysis of variance (not assuming equal variances). Post-hoc analyses were conducted with pairwise t-tests (assuming unequal variance) and pairwise chi-square tests. *p*-values from post-hoc analyses were Bonferroni corrected for comparisons (6) within each variable

Table 1 exhibits NCP had a higher proportion of males than patients with BD2. NCP were generally older than patients with BD1 and BD2. They also had a higher proportion of married individuals and nonsmokers compared to both BD1 and BD2 patients. In addition, their employment rates were higher than those of all the patient groups. They also had a higher level of education and a lower proportion of alcohol users than the MDD and BD2 groups. Furthermore, NCP scored significantly higher on the total ERSQ and CD-RISC measures while having significantly lower Zung-SDS and BAI scores than each patient group (Table 1**)**.

### Factor structure of ERSQ

EFA was conducted on the subsample 1 (*N* = 2,032). The value of the Kaiser-Meyer-Olkin index was *0.96*, indicating that the sample was suitable for factor analysis. A visual inspection of the elbow in the scree plot suggested a one-factor structure. Three eigenvalues were above 1 (14.55, 2.09, 1.32). Based on these results, EFA was performed under one- to three-factor conditions. Smaller RMSR values in models with two or more factors indicated better fit compared to the one-factor model (RMSR = 0.08, 0.05, 0.03 for one-, two-, and three- factor structures, respectively.). However, the third factor of the three-factor model was difficult to interpret clearly. Therefore, the two-factor solution was considered appropriate to parsimoniously extract clearly identifiable factors in both groups.

Similarly, in the analyses conducted on the whole mood disorder patient group (patients with MDD, BD1 and BD2) and the NCP group within subsample 1, two-factor structures were selected following the similar model selection procedure. The results indicated similar factor loading patterns between the two groups. Specifically, the congruence coefficients were *0.97* for both first and second factors, exceeding > 0.95, which suggests a high degree of similarity in factor structures between the groups [51]. The detailed procedure for conducting EFA, along with the factor loadings from the full subsample 1, the whole mood disorder patient group in subsample 1, and the NCP group in subsample 1 based on the two-factor solution, is presented in the Supplementary Note and Tables S1–S3. The factors identified in the EFA seemed reasonable, as factor 1 included items indicating the ability to be aware of and understand emotions and factor 2 included items indicating the ability to tolerate negative emotions and engage in activities.

As the factor loading patterns turned out to be identical between the patient and NCP groups, CFA was employed on subsample 2 (*N* = 2,033). Items with factor loadings < 0.50 in the EFA (items 9, 10, and 15) were deleted. The initial two-factor model suggested by the EFA showed poor fit indices; therefore, modifications were made by inspecting the MIs. Item 2 (“I could consciously bring about positive feelings”) was removed due to covariances suggested by MIs with items 1 (“I paid attention to my feelings”) and 3 (“I understood my emotional reactions”), which loaded on different factors.

**Fig. 1:**
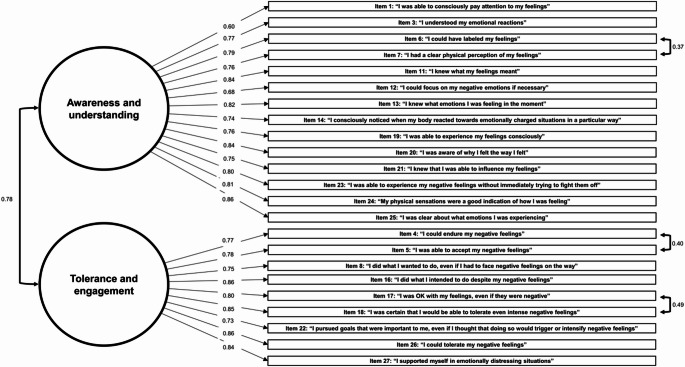
A two-factor model estimated through CFA in subsample 2 (*N* = 2,033). One-way arrows represent factor loadings and two-way arrows represent covariances. All estimates are standardized. CFA, confirmatory factor analysis

Covariances between items loaded on the same factor (items 17 and 18, 4 and 5, 6 and 7, and 11 and 13) were also suggested by the MIs. These covariances were thought to result from the similarities in item meanings and the tendency to respond similarly to adjacent items (e.g., item 6: “I could have labeled my feelings” and item 7: “I had a clear physical perception of my feelings”). Therefore, we added covariances to these items. The fit indices of the final model (Fig. 1) were acceptable (robust CFI = *0.901*; robust RMSEA = *0.093*; and SRMR = *0.044*). Based on the item contents, the two factors were named “awareness and understanding” and “tolerance and engagement.” The “awareness and understanding” reflected the ability to recognize and interpret emotions. The “tolerance and engagement” reflected the ability to remain steadfast in the face of negative emotions and continue moving toward goals. Composite reliability was sufficient for both factors (*ω = 0.95* and *0.93*, for “awareness and understanding” and “tolerance and engagement,” respectively; *ω* for the total composite of the model *= 0.97*). In addition, since the nine-factor structure proposed in the previous English version ERSQ study [20] produced a non-positive definite matrix in our sample, we concluded that the 2-factor model was a better structure for our data. After calculating each subscale score as the average of the items within each factor, the “awareness and understanding” was higher in NCP than in patients with MDD and BD2, and “tolerance and engagement” was highest in NCP and higher in patients with BD1 than in patients with MDD and BD2 (Table 1). The revised ERSQ total score, calculated as the average of the item scores excluding items 2, 9, 10, and 15 based on the result of the factor analysis, was used in subsequent partial correlation and mediation analyses. 

**Fig. 2:**
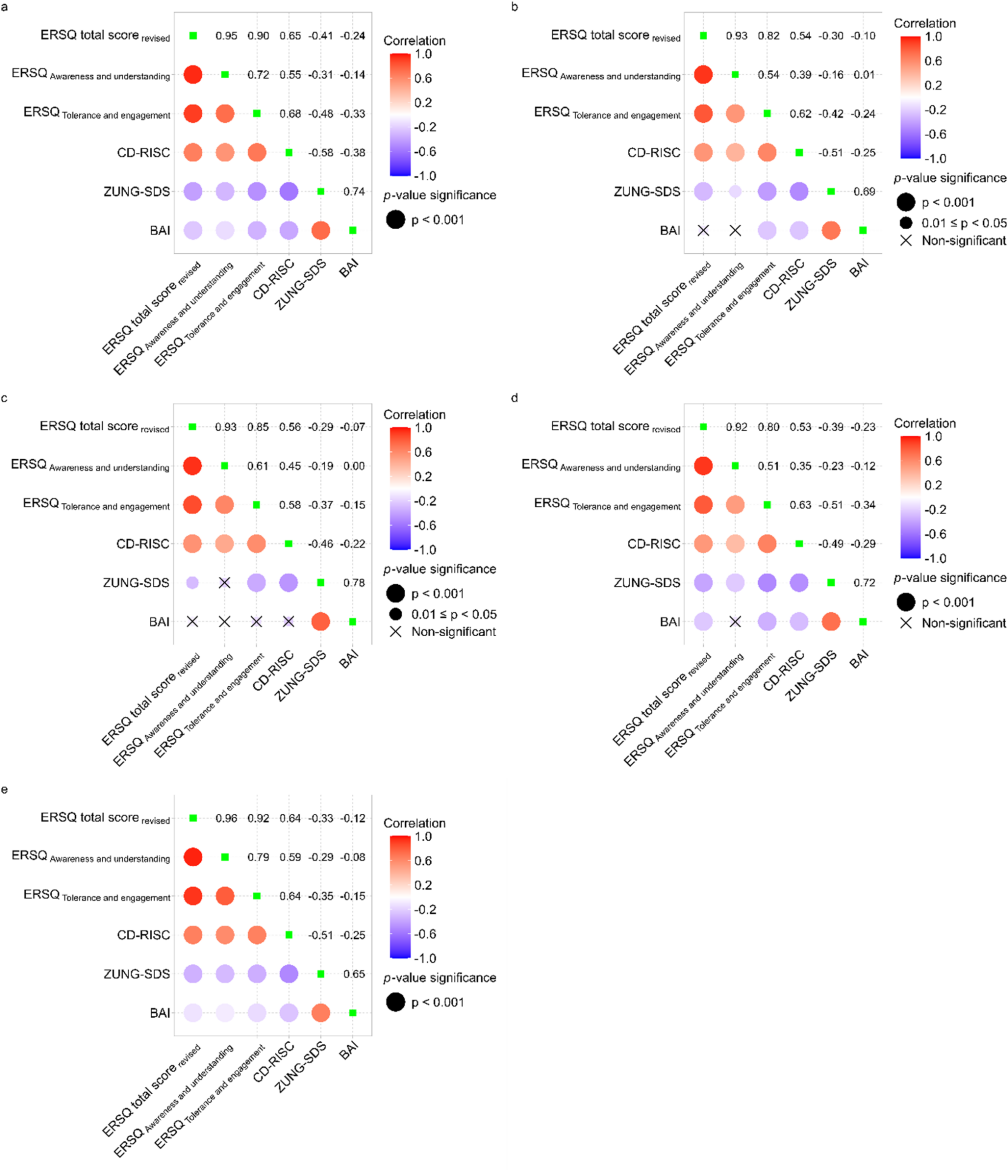
Partial correlations between emotion regulation and resilience, depression symptom severity, and anxiety symptom severity in **a**: the full sample (*N* = 4,065); **b**: MDD (*N* = 362); **c**: BD1 (*N* = 129); **d**: BD2 (*N* = 463); **e**: NCP (*N* = 3,111). Analyses were adjusted for age, sex, education, marital status, job status, alcohol use status, and smoking status. Bonferroni adjustment was applied to correct p-values for each plot. The green boxes on the diagonal represent the standardized variance of each variable, which are equal to 1. ERSQ, Emotion Regulation Skills Questionnaire; ERSQ total score_revised_, ERSQ total score, recalculated with items removed according to the results of the factor analysis; CD-RISC, Connor-Davidson Resilience Scale; Zung-SDS, Zung Self-rating Depression Scale; BAI, Beck Anxiety Inventory

### The relationship between emotion regulation skills and study variables

Figure 2 visualizes the partial correlations among the variables of interest while controlling for potential confounders. The subpanels in Fig. 2 represent the partial correlations for different subgroups: (a) the full sample (*N* = 4,065), (b) MDD (*N* = 362), (c) BD1 (*N* = 129), (d) BD2 (*N* = 463), and (e) NCP (*N* = 3,111). For the total sample presented in Fig. 2a, the total ERSQ score had a significant positive relationship with the CD-RISC score (*r = 0.65*, *p*^*adj*^*<0.001*). Conversely, the total ERSQ score exhibited a significantly negative relationship with the Zung-SDS (*r=-0.41*, *p*^*adj*^*<0.001*) and BAI scores (*r=-0.24*, *p*^*adj*^*<0.001*). Among the subscales of ERSQ, “awareness and understanding” showed significant correlations with CD-RISC, Zung-SDS, and BAI scores (*r = 0.55*, *p*^*adj*^*<0.001*; *r*=-0.31, *p*^*adj*^*<0.001*; *r*=-0.14, *p*^*adj*^*<0.001*, respectively). The “tolerance and engagement” ERSQ subscale was also significantly associated with CD-RISC, Zung-SDS and BAI scores (*r = 0.68*, *p*^*adj*^*<0.001*; *r*=-0.48, *p*^*adj*^*<0.001*; *r*=-0.33, *p*^*adj*^*<0.001*, respectively).

The patterns of the partial correlations in the subgroup analyses were generally consistent with those in the full sample, with a few exceptions. Specifically, in the MDD subgroup, relationships between the ERSQ total score or “awareness and understanding” and BAI were not significant (Fig. 2b). In the BD1 subgroup, the relationships between the BAI, and the total score and the subscales of the ERSQ, were non-significant. In addition, the partial correlation between the “awareness and understanding” subscale of the ERSQ and Zung-SDS was non-significant in the BD1 subgroup (Fig. 2c). In the BD2 subgroup, the partial correlation between “awareness and understanding” subscale and BAI was non-significant (Fig. 2d). All partial correlations in NCP were significant, and the directions of the relationships were identical to those of the full sample (Fig. 2e).

### Mediating role of resilience in the relationship between emotion regulation skills and psychiatric symptoms

**Fig. 3:**
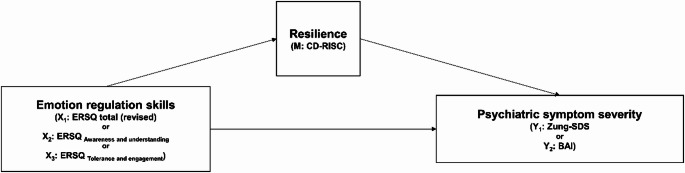
The conceptualized mediation model of the study. Mediation analyses were adjusted for age, sex, education, marital status, job status, alcohol use status, and smoking status. X_i_, total score (recalculated with items removed according to the results of the factor analysis) or subscales of Emotion Regulation Skills Questionnaire (ERSQ); M, Connor-Davidson Resilience Scale (CD-RISC); Y_i_, Zung Self-rating Depression Scale (Zung-SDS) or Beck Anxiety Inventory (BAI)

Figure 3 illustrates the hypothesized mediation relationship between the variables in this study. As shown in Table 2, in the full sample, the indirect effect of emotion regulation skills on both depression and anxiety symptom severity was strong and negative when assessed using the total scale (*β*_*X→M→Y*_*=-*0.345, *p < *0.001; *β*_*X→M→Y*_*=-*0.243, *p* < 0.001, respectively). At the subscale level, the indirect relationships through resilience between each symptom and each factor of emotion regulation skills were also significant and negative (*β*_*X→M→Y*_*=-*0.312, *p < *0.001; *β*_*X→M→Y*_*=-*0.320, *p < *0.001; *β*_*X→M→Y*_*=-*0.227, *p < *0.001; *β*_*X→M→Y*_*=-*0.191, *p < *0.001, corresponding to X_2_ and Y_1_, X_3_ and Y_1_, X_2_ and Y_2_, and X_3_ and Y_2_, respectively). Among the subscales, “tolerance and engagement” showed significant negative direct path estimates for the severity of both depression and anxiety (*β*_*X→Y*_*=-*0.144, *p < *0.001*; β*_*X→Y*_*=-*0.134, *p < *0.001, respectively). Furthermore, the direct path estimate of the “awareness and understanding” was significant and positive for anxiety (*β*_*X→Y*_ *= *0.087, *p < *0.001).

**Table 2:** The mediating role of resilience (M) in the relationship between specific emotion regulation skills (X_i_) and either depression symptom severity or anxiety symptom severity (Y_i_) in the full sample, MDD, BD1, BD2, and NCP, based on the robust maximum likelihood estimator

	M: Resilience	X → M	M → Y	X → M → Y (Indirect)	X → Y (Direct)
Full sample	**Y**_**1**_: **Depression symptom severity**				
(*N* = 4,065)	**X**_**1**_: **ERSQ (revised)**	0.635^***^(0.614, 0.657)	-0.543^***^(-0.576, -0.509)	-0.345^***^(-0.369, -0.321)	-0.049^**^(-0.084, -0.013)
	**X**_**2**_: **Awareness and understanding**	0.535^***^(0.509, 0.560)	-0.583^***^(-0.613, -0.554)	-0.312^***^(-0.333, -0.290)	0.015(-0.018, 0.047)
	**X**_**3**_: **Tolerance and engagement**	0.673^***^(0.653, 0.694)	-0.476^***^(-0.511, -0.440)	-0.320^***^(-0.345, -0.295)	-0.144^***^(-0.181, -0.108)
	**Y**_**2**_: **Anxiety symptom severity**				
	**X**_**1**_: **ERSQ (revised)**	0.635^***^(0.614, 0.657)	-0.383^***^(-0.424, -0.343)	-0.243^***^(-0.271, -0.216)	0.010(-0.030, 0.050)
	**X**_**2**_: **Awareness and understanding**	0.535^***^(0.509, 0.560)	-0.425^***^(-0.463, -0.388)	-0.227^***^(-0.250, -0.205)	0.087^***^(0.048, 0.126)
	**X**_**3**_: **Tolerance and engagement**	0.673^***^(0.653, 0.694)	-0.284^***^(-0.325, -0.243)	-0.191^***^(-0.219, -0.163)	-0.134^***^(-0.174, -0.095)
MDD	**Y**_**1**_: **Depression symptom severity**				
(*N* = 362)	**X**_**1**_: **ERSQ (revised)**	0.528^***^(0.450, 0.606)	-0.491^***^(-0.593, -0.390)	-0.259^***^(-0.323, -0.196)	-0.032(-0.170, 0.105)
	**X**_**2**_: **Awareness and understanding**	0.376^***^(0.282, 0.470)	-0.528^***^(-0.615, -0.441)	-0.199^***^(-0.258, -0.139)	0.046(-0.066, 0.157)
	**X**_**3**_: **Tolerance and engagement**	0.613^***^(0.544, 0.681)	-0.396^***^(-0.508, -0.284)	-0.243^***^(-0.314, -0.171)	-0.183^*^(-0.329, -0.038)
	**Y**_**2**_: **Anxiety symptom severity**				
	**X**_**1**_: **ERSQ (revised)**	0.528^***^(0.450, 0.606)	-0.281^***^(-0.398, -0.163)	-0.148^***^(-0.212, -0.084)	0.050(-0.080, 0.180)
	**X**_**2**_: **Awareness and understanding**	0.376^***^(0.282, 0.470)	-0.304^***^(-0.411, -0.197)	-0.114^***^(-0.163, -0.066)	0.125^*^(0.008, 0.242)
	**X**_**3**_: **Tolerance and engagement**	0.613^***^(0.544, 0.681)	-0.165^**^(-0.290, -0.040)	-0.101^**^(-0.177, -0.025)	-0.142^*^(-0.273, -0.011)
BD1	**Y**_**1**_: **Depression symptom severity**				
(*N* = 129)	**X**_**1**_: **ERSQ (revised)**	0.528^***^(0.393, 0.662)	-0.433^***^(-0.602, -0.264)	-0.229^***^(-0.342, -0.115)	-0.049(-0.261, 0.163)
	**X**_**2**_: **Awareness and understanding**	0.421^***^(0.274, 0.567)	-0.470^***^(-0.620, -0.320)	-0.198^***^(-0.296, -0.100)	0.018(-0.159, 0.194)
	**X**_**3**_: **Tolerance and engagement**	0.560^***^(0.431, 0.688)	-0.372^***^(-0.546, -0.198)	-0.208^***^(-0.323, -0.094)	-0.148(-0.371, 0.075)
	**Y**_**2**_: **Anxiety symptom severity**				
	**X**_**1**_: **ERSQ (revised)**	0.528^***^(0.393, 0.662)	-0.265^**^(-0.455, -0.075)	-0.140^*^(-0.251, -0.029)	0.077(-0.153, 0.307)
	**X**_**2**_: **Awareness and understanding**	0.421^***^(0.274, 0.567)	-0.274^**^(-0.451, -0.098)	-0.115^**^(-0.202, -0.028)	0.114(-0.078, 0.306)
	**X**_**3**_: **Tolerance and engagement**	0.560^***^(0.431, 0.688)	-0.205^*^(-0.395, -0.015)	-0.115^*‡^(-0.228, -0.001)	-0.025(-0.272, 0.221)
BD2	**Y**_**1**_: **Depression symptom severity**				
(*N* = 463)	**X**_**1**_: **ERSQ (revised)**	0.531^***^(0.467, 0.595)	-0.395^***^(-0.487, -0.302)	-0.209^***^(-0.266, -0.153)	-0.182^***^(-0.280, -0.084)
	**X**_**2**_: **Awareness and understanding**	0.347^***^(0.262, 0.431)	-0.469^***^(-0.548, -0.389)	-0.162^***^(-0.211, -0.114)	-0.062(-0.150, 0.027)
	**X**_**3**_: **Tolerance and engagement**	0.650^***^(0.593, 0.706)	-0.272^***^(-0.371, -0.172)	-0.176^***^(-0.243, -0.110)	-0.354^***^(-0.455, -0.252)
	**Y**_**2**_: **Anxiety symptom severity**				
	**X**_**1**_: **ERSQ (revised)**	0.531^***^(0.467, 0.595)	-0.225^***^(-0.333, -0.118)	-0.120^***^(-0.179, -0.061)	-0.116^*^(-0.224, -0.008)
	**X**_**2**_: **Awareness and understanding**	0.347^***^(0.262, 0.431)	-0.279^***^(-0.374, -0.184)	-0.097^***^(-0.137, -0.057)	-0.020(-0.122, 0.082)
	**X**_**3**_: **Tolerance and engagement**	0.650^***^(0.593, 0.706)	-0.119^*^(-0.236, -0.003)	-0.077^*^(-0.153, -0.002)	-0.270^***^(-0.375, -0.165)
NCP	**Y**_**1**_: **Depression symptom severity**				
(*N* = 3,111)	**X**_**1**_: **ERSQ (revised)**	0.645^***^(0.618, 0.671)	-0.507^***^(-0.547, -0.468)	-0.327^***^(-0.356, -0.299)	0.003(-0.037, 0.043)
	**X**_**2**_: **Awareness and understanding**	0.593^***^(0.565, 0.621)	-0.519^***^(-0.556, -0.482)	-0.308^***^(-0.334, -0.282)	0.016(-0.023, 0.055)
	**X**_**3**_: **Tolerance and engagement**	0.640^***^(0.613, 0.667)	-0.488^***^(-0.529, -0.448)	-0.313^***^(-0.341, -0.284)	-0.033(-0.073, 0.007)
	**Y**_**2**_: **Anxiety symptom severity**				
	**X**_**1**_: **ERSQ (revised)**	0.645^***^(0.618, 0.671)	-0.301^***^(-0.346, -0.256)	-0.194^***^(-0.224, -0.164)	0.076^***^(0.035, 0.117)
	**X**_**2**_: **Awareness and understanding**	0.593^***^(0.565, 0.621)	-0.311^***^(-0.355, -0.268)	-0.185^***^(-0.212, -0.157)	0.100^***^(0.060, 0.141)
	**X**_**3**_: **Tolerance and engagement**	0.640^***^(0.613, 0.667)	-0.261^***^(-0.308, -0.215)	-0.167^***^(-0.198, -0.137)	0.015(-0.027, 0.056)

Standardized paths are displayed. ^***^: *p* < 0.001; ^**^: *p* < 0.01; ^*^: *p* < 0.05; ^‡^: *p*-value with borderline significance (*p =* 0.048). 95% confidence intervals are shown in the parentheses. BD, bipolar disorder; MDD, major depressive disorder; NCP, non-clinical participants; ERSQ (revised), ERSQ total score, recalculated with items removed according to the results of the factor analysis

In the MDD subgroup, indirect effect estimates were negative and significant at both the total scale and subscale levels, similar to those observed in the full sample (*β*_*X→M→Y*_*=-*0.259, *p < *0.001; *β*_*X→M→Y*_*=-*0.199, *p < *0.001; *β*_*X→M→Y*_*=-*0.243, *p < *0.001; *β*_*X→M→Y*_*=-*0.148, *p < *0.001; *β*_*X→M→Y*_*=-*0.114, *p < *0.001; *β*_*X→M→Y*_*=-*0.101, *p < *0.01, corresponding to X_1_ and Y_1_, X_2_ and Y_1_, X_3_ and Y_1_, X_1_ and Y_2_, X_2_ and Y_2_, and X_3_ and Y_2_, respectively). Moreover, the “tolerance and engagement” subscale showed significant negative direct path estimates for both depression and anxiety severity (*β*_*X→Y*_*=-*0.183, *p < *0.05*; β*_*X→Y*_*=-*0.142, *p < *0.05, respectively), and the “awareness and understanding” showed a significant positive direct path estimate for anxiety (*β*_*X→Y*_ *= *0.125, *p < *0.05). 

In both BD1 and BD2 groups, the indirect effect to symptom severity through resilience showed a similar pattern to that observed in the entire sample (*β*_*X→M→Y*_*=-*0.229, *p < *0.001; *β*_*X→M→Y*_*=-*0.198, *p < *0.001; *β*_*X→M→Y*_*=-*0.208, *p < *0.001; *β*_*X→M→Y*_*=-*0.140, *p < *0.05; *β*_*X→M→Y*_*=-*0.115, *p < *0.01; *β*_*X→M→Y*_*=-*0.115, *p < *0.05, corresponding to X_1_ and Y_1_, X_2_ and Y_1_, X_3_ and Y_1_, X_1_ and Y_2_, X_2_ and Y_2_, and X_3_ and Y_2_, respectively, in BD1; *β*_*X→M→Y*_*=-*0.209, *p < *0.001; *β*_*X→M→Y*_*=-*0.162, *p < *0.001; *β*_*X→M→Y*_*=-*0.176, *p < *0.001; *β*_*X→M→Y*_*=-*0.120, *p < *0.001; *β*_*X→M→Y*_*=-*0.097, *p < *0.001; *β*_*X→M→Y*_*=-*0.077, *p < *0.05, corresponding to X_1_ and Y_1_, X_2_ and Y_1_, X_3_ and Y_1_, X_1_ and Y_2_, X_2_ and Y_2_, and X_3_ and Y_2_, respectively, in BD2). However, none of the subscales exhibited significant direct path estimates in BD1, while the direct paths from the total emotion regulation skills and “tolerance and engagement” to depression and anxiety severity were significant in BD2 (*β*_*X→Y*_*=-*0.182, *p < *0.001; *β*_*X→Y*_*=-*0.354, *p < *0.001; *β*_*X→Y*_*=-*0.116, *p < *0.05; *β*_*X→Y*_*=-*0.270, *p < *0.001, corresponding to X_1_ and Y_1_, X_3_ and Y_1_, X_1_ and Y_2_, and X_3_ and Y_2_, respectively).

Similar to the patient groups, the indirect effect estimates in NCP were also significant and negative for both symptom severity types (*β*_*X→M→Y*_*=-*0.327, *p < *0.001; *β*_*X→M→Y*_*=-*0.308, *p < *0.001; *β*_*X→M→Y*_*=-*0.313, *p < *0.001; *β*_*X→M→Y*_*=-*0.194, *p < *0.001; *β*_*X→M→Y*_*=-*0.185, *p < *0.001; *β*_*X→M→Y*_*=*-0.167, *p < *0.001, corresponding to X_1_ and Y_1_, X_2_ and Y_1_, X_3_ and Y_1_, X_1_ and Y_2_, X_2_ and Y_2_, and X_3_ and Y_2_, respectively). The direct path was significant only between the total score of emotion regulation skills and anxiety severity and between “awareness and understanding” and anxiety symptom severity, which were positive (*β*_*X→Y*_ *= *0.076, *p < *0.001; *β*_*X→Y*_ *= *0.100, *p < *0.001, corresponding to X_1_ and Y_2_, and X_2_ and Y_2_, respectively). In the bootstrapped 95% confidence intervals presented in **Table S4**, the significance of the estimates was consistent with the results described above.

## Discussion

In this study, we explored the relationships between adaptive emotion regulation skills, resilience, and the severity of depression and anxiety symptoms in patients with mood disorders and non-clinical participants. We conducted partial correlation and mediation analyses based on the factor structure in the Korean version of the ERSQ identified in this study, which included “awareness and understanding” and “tolerance and engagement.” Significant partial correlations were found between the factors of adaptive emotion regulation skills, resilience, and the severity of psychiatric symptoms. Moreover, the mediating roles of resilience were generally identified in the relationships between severity of psychiatric symptoms and the factors of adaptive emotion regulation skills across the total sample, as well as within the BD1, BD2, MDD clinical groups, and non-clinical participants.

Partial correlation analyses showed that resilience was significantly and positively associated with emotion regulation skills, as well as their subscales. These findings were consistent with those of previous studies suggesting that emotion regulation capability is related to resilience [52, 53]. However, emotion regulation skills and resilience showed significantly negative partial correlations with the severity of anxiety and depression, except for several estimates in patients with MDD, BD1, and BD2. Previous studies suggested that emotion dysregulation mediates the influence of childhood trauma on anxiety [54] and the relationship between stressful events and depression [55]. Similarly, resilience mediates the influence of emotional trauma on depression [56], and expects fewer anxiety and depression symptoms following trauma [57]. Hence, our results were consistent with those of previous studies. These relationships are also supported from a neurobiological perspective. Studies suggest that the serotonergic system underpins the mechanisms of both resilience and emotion regulation [58, 59]. Given that the serotonergic system is also associated with anxiety and depression [60], a key link may exist between these clinical features.

As hypothesized, the mediating role of resilience in the effect of emotion regulation skills on psychiatric symptoms was significant in all groups. Regarding the “awareness and understanding” subscale, the size of the indirect effect on anxiety severity was significant; however, the partial correlation between the two variables was insignificant in BD2. Disregarding the possibility of an insufficient sample size, certain factors of resilience influenced by “awareness and understanding” may not be related to anxiety severity, resulting in an overall insignificant contribution of “awareness and understanding” to anxiety severity. Therefore, caution is needed when interpreting this relationship, and further analyses using a factorized resilience scale may help reveal this relationship [61]. The same point also applies to the BD1 group, where the partial correlation between total and subscale scores of emotion regulation skills and anxiety severity, as well as between “awareness and understanding” and depression severity, were not significant.

These findings suggest that helping individuals develop emotion regulation skills can strengthen their resilience [62], which, in turn, may aid in recovery from psychiatric symptoms and in maintaining life with alleviated distress. This is consistent with the results of previous studies suggesting the clinical efficacy of cognitive behavioral therapy (CBT) for depression and anxiety, as CBT can foster emotion regulation [63–65]. Furthermore, our findings suggest that enhancing emotion regulation skills in individuals may be beneficial for controlling life stress, which is related to reduced levels of depression and anxiety symptoms [66, 67].

At the subscale level, some of the direct effects of emotion regulation skills were significant over and above the effect of resilience. Notably, the direct effects of “awareness and understanding” on anxiety severity were significantly positive in the full sample, MDD, and NCP groups. To investigate the underlying reason, we regressed “awareness and understanding” on resilience and sociodemographic variables to calculate the residual. Subsequently, anxiety severity was regressed on the residual, resilience, and sociodemographic variables. Standardized coefficients were calculated using the ‘lm.beta’ package version 1.7-2 [68]. The coefficient of the residual was positive (*β =* 0.07, *p < *0.001 in the full sample, standardized; *β = *0.11, *p < *0.05 in MDD, standardized; *β = *0.08, *p < *0.001 in NCP, standardized). Based on these results, we conjectured that this factor encompasses opposing qualities beyond regulatory emotional awareness. First, “awareness and understanding” may be related to suffering negative emotions, considering the contexts of the items loaded on this factor (e.g., Item 12: “I could focus on my negative emotions if necessary”). This may include rumination, which is associated with anxiety [69, 70]. Second, the emotional sensation perceived with somatization may have contributed to its positive direct effect on anxiety severity [71] (e.g., Item 14: “I noticed it consciously when my body reacted towards emotionally charged situations in a particular way”). However, even when compared to the direct effect, the indirect effect still accounted for a substantial proportion of the total effect, highlighting the crucial mediating role of resilience.

The direct effect of “tolerance and engagement” on psychiatric symptom severities was also significant in the full sample, MDD, and BD2 groups. However, unlike “awareness and understanding,” the estimate was negative. It suggests that the capacity to resist and endure negative feelings has a certain conceptual closeness to depression and anxiety severity, independent of resilience, which represents the general ability to manage stressful circumstances.

Meanwhile, factor analysis of the ERSQ suggested a two-factor structure. The item loading pattern was similar to that of the previous Japanese study [31]. In addition, our result was consistent with a previous network analysis study, which demonstrated that the ERSQ can be divided into two dimensions of recognizing and confronting emotions in patients with MDD [72]. However, the theoretically suggested nine-factor structure [20] seemed to be an inadequate fit for the Korean sample. This may indicate that Koreans do not conceptually differentiate emotion regulation skills as extensively as individuals of other ethnic groups. This difference could stem from cultural influences, such as the Confucian tradition, which is related to a relatively prominent role of non-verbal, less explicit emotional interactions in Korean [73]. Further studies should explore the possible commonalities in factor structure of emotion regulation skills across East Asian countries, which may be contrary to eight- to nine-factor structure studies conducted in Western countries (e.g., Czech, German, English, Turkish, Latvian, Spanish, and French studies) [20, 25–30].

Group differences were observed in the clinical measures, where patients with mood disorder demonstrated lower levels of emotion regulation skills and resilience than NCP. These findings are consistent with those of previous studies indicating that difficulties in emotion regulation are observed in patients with MDD and BD [74, 75] and that resilience mediates the influence of childhood trauma on mood disorders [76]. The patient groups also exhibited higher levels of depression and anxiety severity than NCP, while patients with MDD did not significantly differ from BD2 in emotion regulation skills, resilience, or severity of psychiatric symptoms. Overall, the general trend showed that groups with lower resilience and emotion regulation skills had more severe psychiatric symptoms. However, when comparing inter-group differences, it is important to consider the characteristics of the patient sample. For instance, as the participants were treated in a tertiary medical center, the MDD group contained a substantial proportion of patients with chronic or recurrent courses of depressive symptoms, which may have contributed to lower emotion regulation skills in this group than the values that would have been observed if measured in the general MDD population and may have also contributed to the non-significant difference with the BD2 group [2, 77].

This study has some limitations. First, owing to the cross-sectional nature of the measurements used, causal relationships between study variables could not be established along the temporal axis. Further longitudinal studies are needed to provide evidence supporting our results. Second, the surveys were self-reported, which implies potential methodological confounders such as negative cognitive bias influenced by negative mood [78]. Therefore, measurements rated using objective methods should be incorporated into future research to mitigate these effects. Third, our study did not account for the effects of the current psychosomatic treatment received by patients. As previous studies have suggested, the neurobiological mechanisms underlying resilience and emotion regulation may be influenced by the actions of medical treatments [79, 80], and these factors could be incorporated into further analyses. Fourth, since all participants with mood disorders were recruited from a tertiary care hospital in Korea and the study was conducted exclusively within a Korean population, the generalizability of the findings may be limited. This limitation may reflect Korea-specific factors, such as sociocultural norms shaped by traditional values and biological factors, including gut microbiota influenced by dietary patterns unique to Korean cuisine [81, 82]. Specifically, Korean dietary habits may interact with depression, anxiety, resilience, and emotion regulation through the gut–brain axis [83–85], potentially in culture-bound ways. Therefore, future studies conducted in diverse cultural and clinical settings are warranted to examine whether the observed associations hold across different populations and to clarify the extent to which these findings can be generalized.

Despite these limitations, this study has several strengths. To our knowledge, this was the first study to analyze the relationships between resilience, adaptive emotion regulation skills, and psychiatric symptom severity using a mediation model in patients with mood disorders and non-clinical participants. We also identified the factor structure of the Korean version of the ERSQ and highlighted the role of each factor in the mediation model.

## Electronic supplementary material

Below is the link to the electronic supplementary material.

Supplementary Material 1
