# Supplementary material for: Relationship between emotion regulation skills, resilience, depression and anxiety symptom severity in patients with mood disorders and non-clinical participants: a mediation model
Source: Eur Arch Psychiatry Clin Neurosci. 2025 Jul 3;276(2):459–73. doi: 10.1007/s00406-025-02050-8 (PMC12953268; doi:10.1007/s00406-025-02050-8)
Supplement: Supplementary file 1 — Supplementary Material 1 [file 406_2025_2050_MOESM1_ESM.docx]

**1. Supplementary Note**

**Detailed exploratory factor analysis (EFA) procedure on the whole mood disorder patient group (patients with MDD, BD1 and BD2) and the non-clinical participants in subsample 1**

The value of the Kaiser-Meyer-Olkin index was *0.92* for the whole mood disorder patient group and *0.97* for the non-clinical participant group, both drawn from subsample 1, indicating that the samples were suitable for factor analyses. Visual inspection of the elbow of scree plot suggested one- to two-factor structures in the whole mood disorder patient group and a one-factor structure in the non-clinical participants. The number of eigenvalues above 1 was four in the whole mood disorder patient group (11.65, 3.14, 1.30, 1.23) and three in the non-clinical participants (15.48, 1.57, 1.43). Accordingly, one- to four- factor structures were investigated in the whole mood disorder patient group and one- to three- factor structures for the non-clinical participants. The root mean square of the residual (RMSR) values indicated that models with two or more factors fit better than the one-factor model (RMSR=0.11, 0.06, 0.05, 0.04 for one-, two-, three-, and four-factor structures in the whole mood disorder patient group; RMSR=0.07, 0.05, 0.03 for one-, two-, and three-factor structures in the non-clinical participants). However, as in the full subsample 1, the three-factor solutions in both groups and the four-factor solution in the whole mood disorder patient group were not clearly interpretable. Therefore, two-factor solutions were regarded as suitable for extracting clearly identifiable factors with parsimony in both groups.

**2. Supplementary tables**

**Table S1** Result of exploratory factor analysis with promax rotation on the full subsample 1 (N=2,032).

| **Item No.** | **Factor 1** | **Factor 2** | ***h^2^*** |
| --- | --- | --- | --- |
| 13 | **0.967** | -0.147 | 0.745 |
| 11 | **0.875** | -0.061 | 0.690 |
| 7 | **0.873** | -0.130 | 0.610 |
| 14 | **0.862** | -0.133 | 0.591 |
| 25 | **0.814** | 0.048 | 0.723 |
| 6 | **0.800** | 0.001 | 0.640 |
| 24 | **0.706** | 0.060 | 0.564 |
| 20 | **0.689** | 0.160 | 0.664 |
| 1 | **0.627** | 0.022 | 0.414 |
| 12 | **0.615** | 0.042 | 0.419 |
| 21 | **0.543** | 0.251 | 0.560 |
| 3 | **0.541** | 0.287 | 0.605 |
| 19 | **0.528** | 0.282 | 0.579 |
| 23 | **0.503** | 0.349 | 0.636 |
| 10 | 0.484 | 0.120 | 0.334 |
| 9 | 0.359 | 0.254 | 0.329 |
| 17 | -0.212 | **0.996** | 0.724 |
| 18 | -0.153 | **0.985** | 0.769 |
| 26 | -0.063 | **0.908** | 0.743 |
| 4 | -0.013 | **0.776** | 0.587 |
| 16 | 0.065 | **0.774** | 0.677 |
| 27 | 0.083 | **0.757** | 0.673 |
| 2 | 0.077 | **0.682** | 0.549 |
| 5 | 0.111 | **0.657** | 0.552 |
| 8 | 0.102 | **0.639** | 0.516 |
| 22 | 0.103 | **0.619** | 0.489 |
| 15 | 0.298 | 0.466 | 0.512 |

Factor loadings ≥ 0.50 are indicated in bold.

| **Item No.** | **Factor 1** | **Factor 2** | ***h^2^*** |
| --- | --- | --- | --- |
| 13 | **0.904** | -0.103 | 0.714 |
| 25 | **0.806** | 0.040 | 0.690 |
| 6 | **0.797** | -0.030 | 0.606 |
| 14 | **0.792** | -0.198 | 0.474 |
| 7 | **0.784** | -0.150 | 0.494 |
| 11 | **0.774** | -0.009 | 0.590 |
| 24 | **0.671** | 0.001 | 0.451 |
| 20 | **0.636** | 0.166 | 0.561 |
| 1 | **0.615** | -0.152 | 0.287 |
| 19 | **0.533** | 0.246 | 0.505 |
| 3 | **0.505** | 0.299 | 0.530 |
| 21 | 0.498 | 0.245 | 0.457 |
| 12 | 0.469 | 0.044 | 0.248 |
| 23 | 0.459 | 0.363 | 0.547 |
| 10 | 0.318 | 0.155 | 0.186 |
| 18 | -0.224 | **0.989** | 0.757 |
| 17 | -0.180 | **0.949** | 0.723 |
| 26 | -0.115 | **0.931** | 0.749 |
| 27 | -0.033 | **0.805** | 0.617 |
| 16 | 0.001 | **0.777** | 0.604 |
| 4 | -0.048 | **0.744** | 0.513 |
| 2 | -0.016 | **0.698** | 0.474 |
| 8 | 0.003 | **0.663** | 0.442 |
| 22 | 0.035 | **0.613** | 0.404 |
| 5 | 0.076 | **0.606** | 0.429 |
| 15 | 0.191 | **0.574** | 0.500 |
| 9 | 0.148 | 0.498 | 0.360 |

**Table S2** Result of exploratory factor analysis with promax rotation on the whole mood disorder patient group in subsample 1 (N=456).

Factor loadings ≥ 0.50 are indicated in bold.

Table S3 Result of exploratory factor analysis with promax rotation on the non-clinical participants in subsample 1 (N=1,576).

| **Item No.** | **Factor 1** | **Factor 2** | ***h^2^*** |
| --- | --- | --- | --- |
| 13 | **0.977** | -0.140 | 0.760 |
| 11 | **0.926** | -0.086 | 0.740 |
| 7 | **0.856** | -0.056 | 0.661 |
| 25 | **0.850** | 0.017 | 0.745 |
| 14 | **0.832** | -0.033 | 0.650 |
| 6 | **0.802** | 0.007 | 0.652 |
| 20 | **0.752** | 0.105 | 0.701 |
| 24 | **0.739** | 0.055 | 0.613 |
| 21 | **0.664** | 0.128 | 0.591 |
| 12 | **0.632** | 0.093 | 0.501 |
| 3 | **0.620** | 0.202 | 0.622 |
| 23 | **0.593** | 0.263 | 0.666 |
| 10 | **0.550** | 0.108 | 0.408 |
| 1 | **0.537** | 0.211 | 0.511 |
| 19 | **0.523** | 0.285 | 0.589 |
| 9 | 0.449 | 0.184 | 0.365 |
| 15 | 0.391 | 0.357 | 0.499 |
| 17 | -0.258 | **1.029** | 0.708 |
| 18 | -0.092 | **0.925** | 0.731 |
| 26 | 0.047 | **0.788** | 0.681 |
| 4 | 0.026 | **0.733** | 0.567 |
| 16 | 0.136 | **0.704** | 0.665 |
| 5 | 0.114 | **0.671** | 0.583 |
| 27 | 0.230 | **0.611** | 0.647 |
| 2 | 0.218 | **0.524** | 0.501 |
| 8 | 0.225 | **0.510** | 0.491 |
| 22 | 0.216 | **0.510** | 0.480 |

Factor loadings ≥ 0.50 are indicated in bold.

|  | M: Resilience | X 🡪 M | M 🡪 Y | X 🡪 M 🡪 Y  (Indirect) | X 🡪 Y  (Direct) |
| --- | --- | --- | --- | --- | --- |
| Full sample | **Y_1_: Depression symptom severity** | | | | |
| (N=4,065) | **X_1_: ERSQ (revised)** | 0.635(0.614, 0.657) | -0.543(-0.575, -0.509) | -0.345(-0.369, -0.321) | -0.049(-0.084, -0.013) |
|  | **X_2_: Awareness and understanding** | 0.535(0.509, 0.560) | -0.583(-0.612, -0.553) | -0.312(-0.334, -0.290) | 0.015(-0.018, 0.047) |
|  | **X_3_: Tolerance and engagement** | 0.673(0.652, 0.693) | -0.476(-0.511, -0.441) | -0.320(-0.346, -0.296) | -0.144(-0.181, -0.108) |
|  | **Y_2_: Anxiety symptom severity** | | | | |
|  | **X_1_: ERSQ (revised)** | 0.635(0.614, 0.657) | -0.383(-0.423, -0.343) | -0.243(-0.271, -0.217) | 0.010(-0.030, 0.050) |
|  | **X_2_: Awareness and understanding** | 0.535(0.509, 0.560) | -0.425(-0.462, -0.388) | -0.227(-0.251, -0.205) | 0.087(0.048, 0.125) |
|  | **X_3_: Tolerance and engagement** | 0.673(0.652, 0.693) | -0.284(-0.324, -0.244) | -0.191(-0.219, -0.164) | -0.134(-0.173, -0.095) |
| MDD | **Y_1_: Depression symptom severity** | | | | |
| (N=362) | **X_1_: ERSQ (revised)** | 0.528(0.446, 0.605) | -0.491(-0.590, -0.384) | -0.259(-0.325, -0.197) | -0.032(-0.175, 0.105) |
|  | **X_2_: Awareness and understanding** | 0.376(0.278, 0.468) | -0.528(-0.613, -0.436) | -0.199(-0.260, -0.139) | 0.046(-0.070, 0.156) |
|  | **X_3_: Tolerance and engagement** | 0.613(0.541, 0.680) | -0.396(-0.506, -0.280) | -0.243(-0.315, -0.171) | -0.183(-0.332, -0.039) |
|  | **Y_2_: Anxiety symptom severity** | | | | |
|  | **X_1_: ERSQ (revised)** | 0.528(0.446, 0.605) | -0.281(-0.400, -0.159) | -0.148(-0.215, -0.084) | 0.050(-0.087, 0.181) |
|  | **X_2_: Awareness and understanding** | 0.376(0.278, 0.468) | -0.304(-0.412, -0.192) | -0.114(-0.166, -0.068) | 0.125(0.005, 0.244) |
|  | **X_3_: Tolerance and engagement** | 0.613(0.541, 0.680) | -0.165(-0.291, -0.036) | -0.101(-0.181, -0.022) | -0.142(-0.276, -0.009) |
| BD1 | **Y_1_: Depression symptom severity** | | | | |
| (N=129) | **X_1_: ERSQ (revised)** | 0.528(0.388, 0.663) | -0.433(-0.607, -0.246) | -0.229(-0.362, -0.120) | -0.049(-0.284, 0.169) |
|  | **X_2_: Awareness and understanding** | 0.421(0.267, 0.570) | -0.470(-0.624, -0.307) | -0.198(-0.314, -0.105) | 0.018(-0.179, 0.202) |
|  | **X_3_: Tolerance and engagement** | 0.560(0.426, 0.688) | -0.372(-0.549, -0.181) | -0.208(-0.338, -0.095) | -0.148(-0.382, 0.082) |
|  | **Y_2_: Anxiety symptom severity** | | | | |
|  | **X_1_: ERSQ (revised)** | 0.528(0.388, 0.663) | -0.265(-0.462, -0.060) | -0.140(-0.269, -0.030) | 0.077(-0.178, 0.307) |
|  | **X_2_: Awareness and understanding** | 0.421(0.267, 0.570) | -0.274(-0.456, -0.084) | -0.115(-0.219, -0.032) | 0.114(-0.097, 0.309) |
|  | **X_3_: Tolerance and engagement** | 0.560(0.426, 0.688) | -0.205(-0.399, -0.001) | -0.115(-0.243, -0.0004) | -0.025(-0.291, 0.228) |
| BD2 | **Y_1_: Depression symptom severity** | | | | |
| (N=463) | **X_1_: ERSQ (revised)** | 0.531(0.461, 0.594) | -0.395(-0.485, -0.297) | -0.209(-0.267, -0.153) | -0.182(-0.280, -0.084) |
|  | **X_2_: Awareness and understanding** | 0.347(0.257, 0.429) | -0.469(-0.547, -0.383) | -0.162(-0.214, -0.114) | -0.062(-0.152, 0.025) |
|  | **X_3_: Tolerance and engagement** | 0.650(0.589, 0.702) | -0.272(-0.370, -0.171) | -0.176(-0.245, -0.109) | -0.354(-0.453, -0.252) |
|  | **Y_2_: Anxiety symptom severity** | | | | |
|  | **X_1_: ERSQ (revised)** | 0.531(0.461, 0.594) | -0.225(-0.333, -0.115) | -0.120(-0.180, -0.060) | -0.116(-0.225, -0.006) |
|  | **X_2_: Awareness and understanding** | 0.347(0.257, 0.429) | -0.279(-0.374, -0.180) | -0.097(-0.139, -0.058) | -0.020(-0.128, 0.083) |
|  | **X_3_: Tolerance and engagement** | 0.650(0.589, 0.702) | -0.119(-0.237, -0.001) | -0.077(-0.154, -0.001) | -0.270(-0.377, -0.164) |
| NCP | **Y_1_: Depression symptom severity** | | | | |
| (N=3,111) | **X_1_: ERSQ (revised)** | 0.645(0.618, 0.671) | -0.507(-0.547, -0.468) | -0.327(-0.356, -0.299) | -0.003(-0.043, 0.038) |
|  | **X_2_: Awareness and understanding** | 0.593(0.564, 0.620) | -0.519(-0.556, -0.481) | -0.308(-0.334, -0.282) | 0.016(-0.023, 0.055) |
|  | **X_3_: Tolerance and engagement** | 0.640(0.612, 0.666) | -0.488(-0.528, -0.448) | -0.313(-0.341, -0.284) | -0.033(-0.073, 0.008) |
|  | **Y_2_: Anxiety symptom severity** | | | | |
|  | **X_1_: ERSQ (revised)** | 0.645(0.618, 0.671) | -0.301(-0.346, -0.255) | -0.194(-0.224, -0.163) | 0.076(0.035, 0.117) |
|  | **X_2_: Awareness and understanding** | 0.593(0.564, 0.620) | -0.311(-0.355, -0.267) | -0.185(-0.212, -0.157) | 0.100(0.060, 0.141) |
|  | **X_3_: Tolerance and engagement** | 0.640(0.612, 0.666) | -0.261(-0.308, -0.214) | -0.167(-0.198, -0.137) | 0.015(-0.027, 0.056) |

Table S4 The mediating role of resilience (M) in the relationship between specific emotion regulation skills (X_i_) and either depression symptom severity or anxiety symptom severity (Y_i_) in the full sample, MDD, BD1, BD2, and NCP, based on the maximum likelihood estimator and bootstrapped percentile 95% confidence intervals.

Standardized paths are displayed. Bootstrapped percentile 95% confidence intervals are shown in the parentheses. BD, bipolar disorder; MDD, major depressive disorder; NCP, non-clinical participants; ERSQ (revised), ERSQ total score, recalculated with items removed according to the results of the factor analysis.
